# Expulsion of Tape-Worm by Pumpkin Seeds

**Published:** 1852-11

**Authors:** 


*Expulsion of Tape-Worm by Pumpkin Seeds.*—Case II. Through the politeness of Dr. E. M. Moore, I had an opportunity, about two weeks ago, to administer the pumpkin-seed emulsion to a patient of his, affected with tape-worm. The remedy was taken at 6, A. M., followed by two doses of castor oil, which expelled the worm at noon. When I saw it, it was much corrugated by immersion in strong alcohol, so that its length must have been materially reduced. It measured eighty inches, and was composed of neck, body and tail, but no head. The patient, for a number of years, had expelled fragments of the worm almost daily, and quite recently had pulled off more than a foot in one piece. I proposed another trial of the remedy, with a view of obtaining the *head*, but the experiment has been necessarily postponed. In respect to the evacuation of the head upon which so much stress is laid, it is well to bear in mind, that from the extreme tenuity and fragile nature of the animal's neck, and the means which the head possesses of fastening itself to the bowels, together with the minuteness of the organ itself, various difficulties and disappointments must arise in our efforts to obtain ocular demonstration of this structure. Bremser mentions "an important practical fact, viz., that of the many hundred persons cured by him of tape-worm, not a single individual has seen the head come away."—(Cycl. Pr. Med., art. Worms.)

The seeds used in the above case were of the common pumpkin—cucurbita pepo—and were prepared as follows: Take three ounces of pumpkin seeds, bruise them thoroughly in a mortar, and add water until by expression and straining they afford eight ounces of emulsion. The whole to be taken at once on an empty stomach, followed in two hours by a sufficient purgative dose of castor oil. Cold water to be used freely as a drink, but no food until after the operation.

In Philipp Lorenz Geiger's Hand buch der Pharmacie, Heidelberg, 1829, 2nd vol., p. 1702, the cucurbita occidentalis (West-Indian pumpkin) is recommended, on the authority of D. Mongency, as a valuable remedy for tape-worm.

For *simplicity*, *cheapness* and *safety*, the "pumpkin seed remedy" has no competitor. That its *efficacy* may prove equal to that of kousso and other celebrated articles, is not an unreasonable anticipation, and my object in recording these two cases has been, to induce the readers of the Journal to *test* it and report their experience. W. W. Ely.

Rochester, N. Y., Sept. 27, 1852.—*Boston Med. and Surg. Journal.*

